# Cavity-enhanced magnetic dipole resonance induced hot luminescence from hundred-nanometer-sized silicon spheres

**DOI:** 10.1515/nanoph-2022-0206

**Published:** 2022-07-14

**Authors:** Yi-Chuan Tseng, Sih-Wei Chang, Yang-Chun Lee, Hsuen-Li Chen

**Affiliations:** Department of Materials Science and Engineering, National Taiwan University, No.1, Sec. 4, Roosevelt Road, Taipei 10617, Taiwan; Center of Atomic Initiative for New Materials, National Taiwan University, No.1, Sec. 4, Roosevelt Road, Taipei 10617, Taiwan

**Keywords:** magnetic dipole resonance, phonon-assisted photoluminescence, purcell effect, Si photonics, thin-film cavity

## Abstract

In this paper, we demonstrate the first example of phonon-assisted hot luminescence (PAHL) emission from silicon (Si) spheres (diameter > 100nm) without using the plasmonic effect or quantum confinement effect. Instead, we excite the hot luminescence of Si by a strong thin-film-cavity-enhanced magnetic dipole resonance. The thin-film cavity (80 nm SiO_2_/Ag) shows a strong co-enhancement with the magnetic dipole resonance of Si sphere (diameter = 120 nm). The concentrated electromagnetic fields induce significant light–matter interaction. Our Si sphere coupled with a thin-film cavity achieves a 10-fold field enhancement relative to the Si sphere without an enhancement substrate. Furthermore, we experimentally use cavity-enhanced magnetic dipole resonance to a 50-fold enhancement in PAHL. The measured internal quantum efficiency for the visible light emitted from the Si spheres was approximately 2.4%. Furthermore, we demonstrate the tunability of emission peaks merely by adjusting the sizes of Si spheres using thermal oxidation and etching processes. For comparison, we calculated the peak wavelength (*λ*_peak_) sensitivities (Δ*λ*_peak_/ΔDiameter) of Si spheres and Si QDs through Mie theory and effective mass approximation, respectively. The predicated peak sensitivities of the Si spheres ranged from 1.3 to 3.2; they were much more controllable than those of the Si QDs (200–400). Thus, the peak wavelengths of the PAHL of the Si spheres could be modulated and controlled much more precisely and readily than that of the Si QDs. With the tunability and strong electromagnetic field confinement, the cavity-enhanced magnetic dipole resonance appears to have great potential in the development of all-optical processing based on Si photonics.

## 1 Introduction

Due to the indirect bandgap of silicon (Si), the development of Si-based light-emitting devices for use in future all-optical data processing remains challenging when using Si-based chip technologies. Furthermore, this limitation becomes more critical as the digital data technology industry moves toward the optical interconnections of board-to-board and chip-to-chip communication [1, 2].

To overcome this problem, the strict challenge has been to develop an efficient Si-based light source. Room-temperature visible-light photoluminescence (PL) generated from porous Si has attracted great attention since it was first observed in 1990 [3]. That study inspired many investigations into the origin of PL from porous Si [4–6]. Some experimental studies found a blue-shift of the PL light upon decreasing the size of the porous Si, suggesting the presence of a quantum confinement effect in Si nanostructures [3, 7]. On the scale of quantum confinement, the motion of a randomly moving electron would be restricted to specific and discrete energy levels. When the size of a Si structure reaches quantum-confining dimensions, the energy levels become discrete and, simultaneously, the possibility of luminescent interband recombination is enhanced. Many theoretical [8, 9] and experimental [10–14] studies of quantum confinement effects have been reported for the PL generated from Si nanoparticles and nanocrystals [8–14]. On the other hand, some investigations have suggested that the PL of Si nanocrystals arises from radiative recombination at defect states [13, 15]. Localized defect states and the quantum confinement of excitons in Si nanocrystals have both been regarded as possible mechanisms for the PL phenomenon from Si nanostructures [16]. With improvements in the techniques of nanoscale fabrication and the preparation of defect-free quantum dots (QDs) [17], attempts have been made to emit light from Si QDs in a spectral range from the ultraviolet (UV) to the near infrared (NIR) [18]. Furthermore, it is possible, by alloying Si and Ge, to convert the nature of Si structures from having an indirect band gap to having a direct band gap [19–21]. For example, Si_1–*x*_Ge_*x*_ (*x* > 0.65) alloy nanowires have a direct band gap [19], opening up new routes for inducing efficient PL from Si. Nevertheless, the fabrication of high-quality hexagonal SiGe nanowires requires a single-crystal III–V semiconductor (GaAs [19] or GaP [21]) as the lattice-matched core, and hexagonal SiGe nanowires exhibit limited NIR emission of the photon energy only in the regime from 0.3 to 0.6 eV.

Typically, the methods for tuning the spectral ranges of light emitted from Si structures displaying quantum confinement effects have been based on precise control of the sizes of Si QDs or nanostructures. Although the PL spectra could be tuned readily by varying the size of the Si QDs, precise control over the diameter of Si nanostructures on sub-5 nm scale is a challenge in current semiconductor fabrication techniques of electronic devices. Although the bottom-top method of Si QDs synthesis can control the size precisely on sub-5 nm scale, the synthesis of QDs are difficult to be integrated with integrated circuit (IC) fabrication and conventional optoelectronic devices. Therefore, the challenge remains to develop Si-based lighting structures with features on a larger scale—for example, on the order of hundred nanometers—where the quantum confinement effect cannot be exploited.

To achieve the emission of light from Si structures having dimensions on the scale of several tens of nanometers, Cho et al. applied a non-thermalized carrier recombination mechanism to couple Si nanowires with plasmonic nanocavities [22]. They demonstrated that visible light could be emitted from a Si nanowire of several tens of nanometers when integrated with an Ω-shaped plasmonic nanocavity by exploiting the Purcell effect [23]. The concentrated electromagnetic fields inside the plasmonic nanocavity induced the emission of phonon-assisted luminescence from the hot carriers prior to their thermalization to the lowest energy state in the conduction band. That study was the first to overcome the limitations of PL light being emitted only from Si structures on the quantum-confinement scale. Nevertheless, the need for noble metal nanocavities increases the complexity of the fabrication process and increase the non-radiative loss due to the optical absorption of metal.

To readily emit light without a quantum confinement effect, in this study we utilized the magnetic dipole (MD) resonance of Si sphere to induce the emission of visible light. In 1983, Kerker et al. theoretically predicted an MD resonance effect from dielectric particles. They proposed that hypothetical magneto-dielectric particles exhibiting both electric and magnetic resonances with coherent effects would generate strong electromagnetic resonant phenomena [24]. Resonant light scattering phenomena generated from high-refractive-index dielectric and semiconductor nanoparticles have been demonstrated experimentally through magnetic resonance at optical and infrared frequencies [25–28]. Si particles having large refractive index could, therefore, support strong MD resonances [29]. Zheng et al. reported that strong MD resonance could greatly enhance two- or three-photon absorption–induced visible PL in Si spheres [30]. Furthermore, Xiang et al. found that GaAs nanospheres having a large refractive index (*n* = ca. 4) could emit strong second harmonic generation signals through the assistance of MD resonance [31]. These studies confirmed the electromagnetic field enhancement effect of magnetic resonance in materials of high-refractive index.

In this study, we observed visible light emission from Si spheres without exploiting a quantum confinement mechanism, but with the assistance of a strong MD resonance effect. The strong displacement current driven by the MD resonance highly increases the field confinement inside the Si sphere. To further enhance the electromagnetic field, we prepared a thin-film cavity and coupled the Si spheres with the cavity system. Unlike the Si nanostructure coupled with plasmonic cavity, we utilize a thin-film cavity with a spacer having thickness of several tens of nanometers. The thin-film cavity greatly enhanced the MD resonance and provided an intense electromagnetic field in each Si sphere. Without using a plasmonic cavity, the non-radiative loss due to the absorption of metal is highly decreased. Moreover, to verify that MD resonance in the Si spheres could induce the emission of phonon-assisted hot luminescence (PAHL), we used three-dimensional finite-difference time-domain (3D-FDTD) simulation to analyze the spectral resonance of the electromagnetic field within the Si spheres. Furthermore, we experimentally tuned the resonant size of the Si spheres through precisely controlled thermal oxidation and etching processes. The Si spheres coupled with thin-film cavity provide a tunable visible light emission by controlling the size. The MD resonance of Si sphere can be used to induce the PAHL from Si spheres on the non-quantum-confinement scale.

## 2 Results and discussion

**Figure 1:**
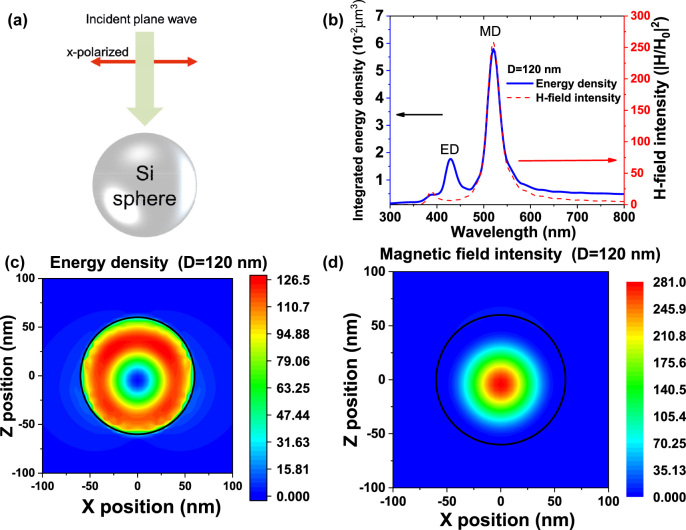
(a) Schematic representation of a freestanding Si sphere illuminated by plane wave. (b) Simulated integrated energy density and magnetic field intensity (|*H*/*H*_0_|^2^) of a freestanding Si sphere (*D* = 120 nm). (c, d) energy density distributions (c) and magnetic field intensity in the *x*–*z* plane of the Si sphere at wavelength of 521 nm.

To study the MD resonance from Si spheres, we firstly simulated the behavior of field enhancement in a single Si sphere. We used the 3D-FDTD approach to simulate the behavior of electromagnetic field in a freestanding Si sphere (diameter: 120 nm). Figure 1(a) displays a schematic representation of our simulation model: a x-polarized plane wave source is applied and launch plane wave to a free-standing Si sphere having a diameter of 120 nm. Figure 1(b) displays the integrated energy density ( $\left\langle \rho \right\rangle$ ) and magnetic field intensity (|H|^2^/|H_0_|^2^) of a freestanding Si sphere. The integrated energy density shows a similar peak position with the magnetic field intensity. The peak position of integrated energy density is located at wavelength of 521 nm, which is corresponding to the peak position of MD mode predicted by Mie theory [32]. The correlation between integrated energy density and magnetic field intensity indicates that the strong potential of MD resonance to enhance the light emission from a Si sphere. Figure 1(c) and (d) further depict the spatial distribution of energy density and magnetic field intensity in a Si sphere at wavelength of 521 nm, respectively. The energy density displays a circular pattern and a maximum value of approximately 126 can be obtained. The magnetic field intensity performs a hot spot at the center of the Si sphere and a maximum value of magnetic field intensity is 281. The circular pattern of energy density can be interpreted as the strong displacement current loop driven by the MD resonance. In addition, the integrated energy density also shows a small peak, which is induced by the electric dipole (ED) resonance, at wavelength of 430 nm. However, ED resonance provides a weaker electromagnetic field confinement and a lower integrated energy density. The spatial distribution of energy density shows that the maximum value of 111.5 (Supplementary Material, S1), which is smaller than that of the MD resonance. Moreover, the hotspot area is much smaller than the MD resonance one, which shows a circular electric hot zone driven by strong magnetic dipole resonance in a Si sphere.

**Figure 2:**
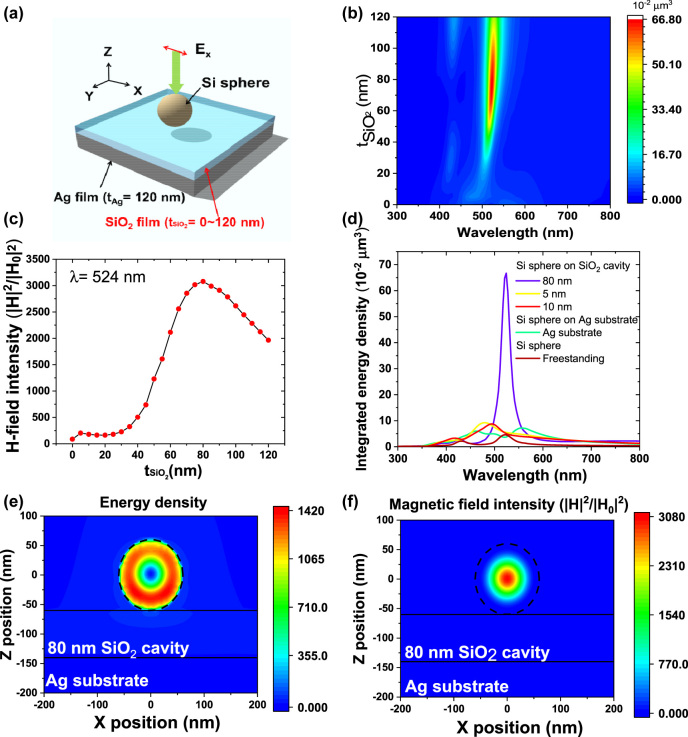
(a) Schematic representation of a Si sphere on a thin-film cavity substrate having a SiO_2_ film of various thicknesses, used to simulate the energy density and magnetic field intensities inside the Si sphere. *t*_SiO2_ is the thickness of SiO_2_. (b) Contour map of integrated energy density in the Si sphere with various thickness of the SiO_2_ layer in the thin-film cavity substrate. (c) Calculated magnetic field intensities at the center of the Si sphere plotted with respect to the thickness of the SiO_2_ layer in the thin-film cavity substrate. (d) Integrated energy density in Si sphere with various substrate. (e, f) Calculated energy density (e) and magnetic field intensity (f) of 120 nm Si on the 80 nm SiO2 thin-film cavity.

With the high refractive index, Si sphere can support strong MD resonance and confine strong electromagnetic fields. To further investigate and increase the field enhancement effect of Si sphere, we designed a thin-film cavity to further increase the light–matter interaction in Si sphere via 3D-FDTD method. Figure 2(a) displays a schematic representation of the simulation model: an x-polarized plane source was applied incident to Si sphere (diameter: 120 nm) placed upon a thin-film cavity having a SiO_2_ film thickness in the range from 0 to 120 nm deposited on an Ag substrate. The simulation result of integrated energy density with various thickness of SiO_2_ layer is shown in Figure 2(b). Due to the interference effect of the thin-film cavity, when the SiO_2_ layer has a thickness of 80 nm, the integrated energy density shows a maximum value of 66.8 × 10^−20^ μm^3^, which is approximately 1050% enhancement relative to the freestanding Si sphere. Furthermore, as shown in Figure 2(c), the magnetic field intensity at the center of Si sphere also shows a maximum value when the SiO_2_ layer has thickness of 80 nm. The magnetic field intensity shows a maximum value of 3080, which is approximately 1000% enhancement compared with the freestanding case. The highly positive correlation between energy density and magnetic field intensity is significant. It is worth to be noted that our thin-film cavity focuses on the interference effect rather than the plasmonic effect of the metallic substrate, which is quite different from the previous studies about Si nanostructure coupled with an ultra-thin nanospacer [33–35]. Although an ultra-thin nanospacer can enhance the electric field efficiently at the gap between the Si sphere and the metallic layer through the near-field plasmonic effect, the electric field is not well confined inside the Si sphere but only at the gap. Figure 2(d) shows the integrated energy density in the Si sphere on various substrates. The 80 nm thin-film cavity shows a highest energy density. Lower values of integrated energy density are observed for Si spheres placed on Ag substrate, 5 nm SiO_2_ coated Ag substrate and 10 nm SiO_2_ coated Ag substrate. Even with the plasmonic effect of Ag substrate coupled with Si sphere, the integrated energy densities display a much lower values than the 80 nm thin-film cavity. With these results, a plasmonic nanocavity cannot concentrate the light field inside the Si sphere effectively. Figure 2(e) and (f) display the spatial distribution of energy density and magnetic field intensity of Si sphere placed on the 80 nm thin-film cavity. The simulated energy density distribution shows a strongly confined electric field inside the Si sphere (Figure 2(e)). A strong magnetic hotspot inside the Si sphere is also confirmed in the results (Figure 2(f)). The electromagnetic field confinement reached an approximately 10-fold enhancement compared with the freestanding Si sphere (Figure 1(c) and (d)).

**Figure 3:**
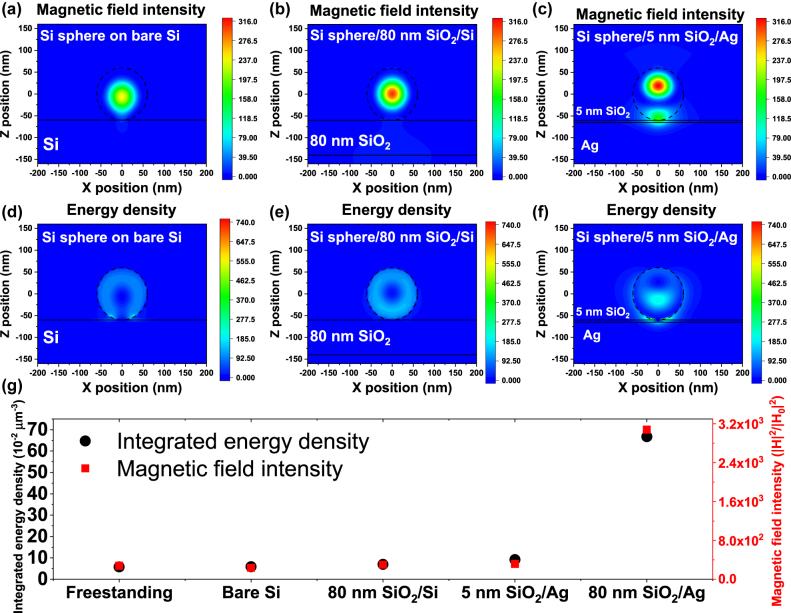
(a–c) Magnetic field intensity and (d–f) energy density distributions in Si spheres on various substrates: (a, d) on bare Si, (b, e) 80 nm SiO_2_/Si (c, f) 5 nm SiO_2_ thin-film cavity. (g) Integrated energy densities and magnetic field intensities of Si spheres on various substrates.

To further compare the electromagnetic field enhancement on various substrates, we simulated the energy density and magnetic field intensity in Si spheres placed on various substrates, including bare Si, 80 nm SiO_2_ coated on Si, and 5 nm SiO_2_ layer coated on Ag. Figure 3(a) and (d) shows the simulated energy density and magnetic field intensity of Si sphere on bare Si, respectively. The energy density shows a circular pattern and a hotspot near the interface between the Si sphere and bare Si. The confined electromagnetic field of Si on bare Si is weaker than the freestanding case (Figure 1(c) and (d)). This decrement may come from the waveguide modes of bare Si which weaken the field confinement in the Si sphere. To confirm the interference effect from the thin-film cavity, we simulated the electromagnetic field distribution of Si sphere placed on 80 nm SiO_2_ coated on Si substrate (80 nm SiO_2_/Si). As shown in Figure 3(b) and (e), the energy density and magnetic field intensity are both slightly higher than those of the freestanding case. The enhancement comes from the interference effect of the SiO_2_ layer and Si substrate, but the enhancement is much weaker than the optimized thin-film cavity (80 nm SiO_2_/Ag). These simulation results confirm that the interference effect plays an important role in the thin-film cavity structure. Next, we show the energy density and magnetic field intensity distribution of Si sphere placed on 5 nm SiO_2_ coated on Ag substrate, which is a common configuration for coupling plasmonic metal and Si sphere (Figure 3(c) and (f)). The energy density distribution shows a strong hot spot near the interface of Si sphere and 5 nm SiO_2_ layer and a maximum energy density of 740 can be observed in the gap between Si sphere and Ag substrate. However, the electromagnetic field confined in the thin-film spacer is not suitable for enhancing the light–matter interaction inside the Si sphere. Moreover, we found that the optimized thin-film cavity provides a much stronger maximum energy density of 1420 in the sphere (Figure 2(c)). These results indicate that a cavity-enhanced MD resonance can confine more light into the Si sphere than a plasmonic cavity. A plasmon based electromagnetic concentration method is suitable for a light emitter with a nanometer-sized light emitter (e.g. quantum dots) due to the extremely small wavelength of surface plasmon. For a hundred-nanometer-sized Si sphere, the light field confinement in the whole volume of Si sphere is highly crucial. A global electric field confinement by cavity-enhanced MD resonance is more effective than a localized confinement of the plasmonic effect. Thus, energy density of plasmonic configuration is lower integrated than the optimized thin-film cavity one. Figure 3(g) summarized the integrated energy intensity and magnetic field intensity. The optimized 80 nm thin-film cavity shows a significantly stronger field enhancement effect and can be a superior potential platform for enhancing the PAHL.

**Figure 4:**
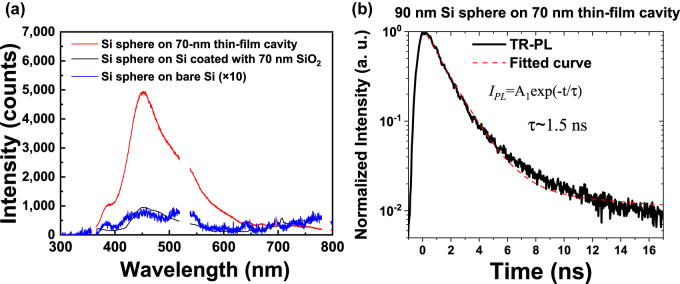
(a) Emission spectra of Si spheres (diameter: 90 nm) placed upon the various substrates. (b) Time-resolved PL intensity of Si spheres (diameter: 90 nm) placed upon a thin-film cavity.

**Figure 5:**
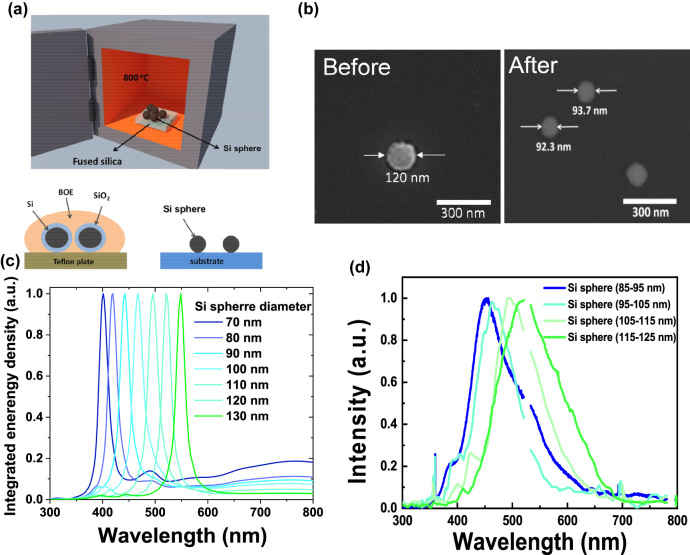
(a) Schematic representation of process used to decrease the size of the Si spheres. (b) Top-view SEM images of resulting Si spheres before and after the process. The size of Si sphere before and after the process is approximately 120 nm and 90 nm, respectively. (c) Simulated integrated energy density of Si sphere having various diameters placed on the thin-film cavity. (d) Experimental emission spectra of Si spheres having diameters of ca. 90, 100, 110, and 120 nm.

To confirm the field enhancement effect of Si sphere coupled with thin-film cavity, we experimentally excited the PAHL of Si sphere by using a *µ*-PL microscope. We used a 266 nm laser as the excitation source to generate PAHL from Si spheres with an objective lens having NA = 0.23 (see Methods). With a microscope system, the incident laser may contain an oblique component, which is different from the simulation in the previous section. The detailed discussion about the thin-film cavity under oblique incidence is shown in the Supplementary Material S4. Figure 4(a) shows the measured PL intensity from Si sphere on 70 nm SiO_2_/Ag, Si sphere on 70 nm SiO_2_/Si, and Si sphere on bare Si. The diameters of Si spheres are ca. 90 nm (Figure 5(b)). The PAHL shows a peak at the wavelength of around 452 nm, which is dominated by MD resonance. By utilizing a 70 nm SiO_2_ thin-film cavity, the PAHL intensity in Si sphere is greatly enhanced by the coupling between MD resonance and thin-film cavity. The spacer of 70 nm SiO_2_ layer is according to the simulation of a Si sphere having diameter of 90 nm (Supplementary Material, S2). The integrated peak area of Si sphere on thin-film cavity is around 5 times higher than the peak area of Si sphere on 70 nm SiO_2_ layer coated on bare Si. Furthermore, the PAHL intensity of Si sphere on bare Si is weak and the peak area of Si sphere on thin-film cavity is around 50 times of the Si sphere on bare Si. The thin-film cavity appeared to be a superior substrate for enhancing the light emission from Si spheres, due to it greatly enhancing the MD resonance of the Si spheres. To further investigate the PAHL, we used time-resolved PL to characterize the radiative decay rate of Si sphere. The time-resolved PL spectrum in Figure 4(b) reveals that the Si spheres (diameter: 90 nm) placed upon the 70 nm thin-film cavity structure had a lifetime of 1.5 ns. This lifetime is corresponding to a typical value of phonon-assisted exciton recombination (1–10 ns) in a “bulk-sized” Si (no quantum-confinement) [36].

Because the spectral position of the MD resonance inside the Si sphere could be tuned readily by adjusting the size of the Si spheres, we applied thermal oxidation and wet etching processes to the Si spheres to decrease their diameter. Figure 5(a) provides a schematic representation of these processes; details are described in the Methods section. Figure 5(b) displays SEM images of the Si spheres obtained before and after 15 h of thermal oxidation and etching; the diameter of the Si spheres had decreased to approximately 90 nm from 120 nm. Hence, we have demonstrated an ability to tune the peak positions of the hot luminescence from the Si spheres. As mentioned above, because the MD resonance of the Si sphere played an important role in inducing phonon-assisted luminescence, a simple strategy for adjusting the peak positions involves varying the diameter of the Si sphere. We also used 3D-FDTD method to simulate the integrated energy density of Si sphere on thin-film cavity with various diameters. Figure 5(c) presents the simulated integrated energy density of Si spheres having diameter from 70 to 130 nm. Blue-shifting of the main peak occurred, from 548 to 400 nm, upon decreasing the diameter of the Si sphere, presumably because of the corresponding blue-shifted MD resonance. We also experimentally verify our ability to tune the peak positions of the phonon-assisted luminescence generated from the Si spheres. We placed Si spheres having diameters ranging from 85 to 125 nm (after applying the thermal oxidation and wet etching processes) upon the Ag/SiO_2_ thin-film cavity substrate and observed their light emission spectra. Figure 5(d) reveals that the Si sphere having a diameter of approximately 120 nm provided a broad emission signal with its peak located near 520 nm; the PAHL emission peaks blue-shifted to 494, 462, and 452 nm upon decreasing the size of the Si spheres to approximately 110, 100, and 90 nm, respectively. Although the peak positions of the measured light emission spectra matched the simulated results well, the experimental signals were broader than the predicted ones. We attribute this broadening of the emission peaks to two factors: (1) the Si spheres had some differences in their shapes and size distributions and (2) the excited carriers would emit photons with relatively continuous frequencies as a result of phonon-assisted recombination during relaxation through intraband thermalization. The detailed discussions about the shape effect and size distribution are shown in the Supplementary Material S5 and S6.

**Figure 6:**
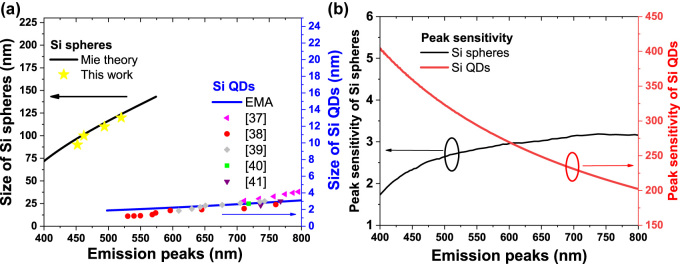
(a) Emission peaks of Si spheres (this study) of various sizes compared with those reported previously for Si QDs [37–41]. (b) Peak sensitivities of Si QDs and Si spheres, calculated using EMA and Mie theory, respectively.

We have demonstrated that the PAHL from Si spheres has a potentially tunable emission wavelength. To further investigate the potential of tuning the emission wavelength of Si spheres, we compared the emission peaks of our Si spheres with those from previous studies of light emission from Si QDs. As displayed in Figure 6(a), our Si spheres exhibited blue to green light emission with wavelengths ranging from 450 to 550 nm. In the previous studies, the emission peaks of the Si QDs were also changed by tuning their size, with wavelengths ranging from 550 to 800 nm [37–41]. Although there have been some studies of blue light emission from Si QDs having the size of less than 1.5 nm, the extremely high surface area–to–volume ratio of such Si QDs makes them difficult to control the processes. To further characterize and compare the peak tunability of the Si spheres and Si QDs, we define the peak sensitivity as (Δ*λ*_peak_/Δ*D*), where Δ*λ*_peak_ is the wavelength of the emission peak and *D* is the size of the Si spheres or QDs. Table S1 summarizes the positions of the emission peaks and their peak sensitivities. The experimental peak sensitivities of the Si QDs in previous studies have ranged from 24.50 to 333.3; these values are much larger than the peak sensitivities of the Si spheres in this present study, ranging from 1.0 to 4.3. There is difficulty in controlling the peak position of the light emitted from Si QDs because it requires precise control over the size of the Si QDs. To further compare the peak tunability, we used Mie theory and the effective mass approximation (EMA) to calculate the peak sensitivities of the Si spheres and Si QDs, respectively (Figure 6(b)). The predicated peak sensitivities of the Si spheres ranged from 1.3 to 3.2, much lower than the range for the Si QDs (200–400). Furthermore, in our present study, the size of the Si spheres could be tuned reliably through the application of simple thermal oxidation and etching processes. The emission peak could be tuned well simply through variation of the size of the Si sphere. Thus, the peak wavelength of the PAHL from the Si spheres could be modulated and controlled more precisely and readily than that from the Si QDs.

## 3 Conclusions

In this study, we have demonstrated that the strong electromagnetic fields confinement induced from cavity-enhanced MD resonance can be used to induce PAHL from Si spheres having hundred-nanometer size. The concentrated electromagnetic fields that result from cavity-enhanced MD resonance strongly increase the light–matter interaction. Following on the 3D-FDTD simulations, we observed a 10-fold field enhancement in Si sphere coupled with thin-film cavity compared with a freestanding one. In addition, we compare the electromagnetic field confinement ability between our thin-film cavity and plasmonic nanocavity. The thin-film cavity enhanced Si sphere shows a much stronger field confinement effect inside the Si sphere than a plasmonic nanocavity. In addition, we used thermal oxidation and etching processes to accurately control the sizes of the Si spheres. The internal quantum efficiency of the visible light emission from the Si spheres was approximately 2.4%. This study provides evidence that the strong electromagnetic fields resulting from thin-film cavity–enhanced MD resonance can assist in inducing hot light emission from “bulk-sized” Si spheres (no quantum confinement effect). To the best of our knowledge, the emission of visible light from hundred-nanometer-sized Si spheres without utilizing plasmonic effect has not been reported previously. Moreover, previous studies of Si QDs have revealed a difficulty in controlling the peak position, requiring precise control over the sizes of the Si QDs. In this study, however, we have found that the peak positions of the emissions from Si spheres can be tuned readily merely by adjusting the size of the Si spheres. For comparison, we used Mie theory and the EMA to calculate the peak sensitivities (Δ*λ*_peak_/Δsize) of Si spheres and Si QDs, respectively. The predicated peak sensitivities of the Si spheres ranged from 1.3 to 3.2; they were much more controllable than those of the Si QDs, which had the peak sensitivities ranging from 200 to 400. Thus, the peak wavelengths of the hot luminescence from Si spheres can be modulated and controlled much more precisely and readily than those of Si QDs. Therefore, we suggest that this concept of thin-film cavity–enhanced MD resonance should be very applicable in the development of next-generation techniques for all-optical processing by Si photonics.

## 4 Methods

### 4.1 Sample preparation

Si spheres (diameter: ca. 120 nm; Emaxwin Technology) were dispersed (concentration: 5 × 10^−3^ wt%) in ethanol through ultrasonication for 30 min. The suspension of Si spheres was then spun onto substrates at a spinning rate of 3000 rpm. To investigate and enhance the electromagnetic field inside the Si spheres, three kinds of substrates were prepared for comparison: a bare fused silica substrate, a Si substrate capped with 70 nm SiO_2_ (Si/70 nm SiO_2_), and a Si substrate capped with metal/dielectric (Si/120 nm Ag/70 nm SiO_2_) thin-film based cavity. The fused silica and Si substrates were cleaned sequentially with acetone, isopropyl alcohol (IPA), and deionized water and then dried under a flow of N_2_. The Si/70 nm SiO_2_ substrate was prepared through sputtering of a SiO_2_ film (thickness: 70 nm) onto a pre-cleaned Si substrate. The thin-film cavities were prepared by sputtering Ag films (thickness: 120 nm) onto cleaned Si substrates, and then sputtering dielectric spacers (SiO_2_) onto the metal films. To decrease the size of the Si spheres through thermal oxidation and wet etching processes, the following processes were applied: (i) Si spheres were placed on a fused silica substrate and heated in a furnace at atmospheric pressure, to thermally grow a SiO_2_ shell on the surface of the Si spheres. The Si spheres were heated to 800 °C at a rate of 20 °C/min; the duration of the oxidation process was varied from 5 to 15 h to adjust the size of the Si spheres by adjusting the thickness of the thermally formed SiO_2_ shell. (ii) The Si spheres with SiO_2_ shells were transferred to a Teflon substrate and the SiO_2_ shells were etched in a buffered oxide etch (BOE) solution (40%) for 10 min. (iii) The remaining shrunken Si spheres were transferred onto the various substrates for further study.

### 4.2 Sample characterization

The Si spheres were observed using scanning electron microscopy (SEM, NOVA NANO 450). The light emission spectra of the Si spheres samples were recorded using a commercial *µ*-PL microscope (UniRAM, UniNanoTech) equipped with a monochromator having a focal length of 20 cm. The wavelength of the excitation laser UV light was fixed at 266 nm (Nd:YAG laser). The PL data at around 532 nm has been excluded due to the strong 2nd harmonic signal from the Nd:YAG laser. The laser beam was focused by a 10× objective having a numerical aperture of 0.23. The spot size of the excitation laser was approximately 6.25 μm^2^. Raman spectra of the Si spheres were recorded using a commercial micro-Raman microscope (UniRAM, UniNanoTech) equipped with a monochromator having a focal length of 75 cm. The wavelength of the excitation laser line was fixed at 532 nm (diode laser). The laser beam was focused by a 100× objective having a numerical aperture of 0.95. The spot size of the excitation laser was approximately 0.4 μm^2^.

### 4.3 Quantum efficiency estimation

The quantum efficiencies of the Si spheres were estimated after placing them on the designed thin-film cavity and comparing their emission behavior with that of common organic dyes; this method has been used in previous studies [42, 43]. The relationship between the light emission and the pump excitation power could be described by the relation *P*_PAHL_ ∝ *η*_a_ *η*_c_ *η*_q_ *P*_ex_, where *P*_PAHL_ and *P*_ex_ are the phonon assisted enhanced hot emission and excitation powers, respectively, and *η*_a_, *η*_c_, and *η*_q_ represent the absorption of the material, the collection efficiency of the light emission, and the quantum efficiency, respectively. The absorption of rhodamine 6G was measured at a wavelength of 266 nm and then the 3D-FDTD approach was used to simulate the absorption of a Si sphere. The 3D-FDTD calculations were performed for a Si sphere placed on a designed thin-film cavity (thickness: 70 nm) and for rhodamine 6G on a fused silica substrate, to obtain the overall far-field outcoupling efficiencies. (These calculations considered the numerical aperture of the objective.) Finally, by comparing the light emission power from the Si sphere and from rhodamine 6G, the quantum efficiency of Si spheres could be estimated.

### 4.4 Energy density and magnetic field intensity calculation

In this paper, we use energy density to estimate the electric field enhancement in the Si sphere. We perform the 3D-FDTD simulations to calculate the energy density in the Si sphere coupled with thin-film cavity. The integrated energy density over the volume of Si sphere can be calculated directly by the relationship:

(1) ρ=∫vsiReεE2E02dV,

where Re[*ɛ*] is the real part of the complex dielectric constant, |*E*|^2^/|*E*_*0*_|^2^ is the electric field normalized by the incident field, and Re[*ɛ*]|*E*|^2^/|*E*_*0*_|^2^ can be defined as the energy density. The magnetic field intensities of Si spheres are collected by using a steady-state monitor at the center of the Si sphere.

## Supplementary Material

Supplementary Material Details
