# Supplementary material for: Cavity-enhanced magnetic dipole resonance induced hot luminescence from hundred-nanometer-sized silicon spheres
Source: Nanophotonics. 2022 Jul 14;11(16):3583–93. doi: 10.1515/nanoph-2022-0206 (PMC11501341; doi:10.1515/nanoph-2022-0206)
Supplement: Supplementary file 1 — Supplementary Material Details [file j_nanoph-2022-0206_suppl.docx]

**Supplementary Material**

Cavity-enhanced Magnetic Dipole Resonance Induced Hot Luminescence from Hundred-nanometer-sized Silicon Spheres

Yi-Chuan Tseng, ^a††^ Sih-Wei Chang, ^a††^ Yang-Chun Lee, ^a^ and Hsuen-Li Chen ^*a b^

1. Department of Materials Science and Engineering, National Taiwan University, No.1, section 4, Roosevelt Road, Taipei, 10617, Taiwan
2. Center of Atomic Initiative for New Materials, National Taiwan University, Taiwan
3. E-mail: hsuenlichen@ntu.edu.tw; Tel: +886 2 3366 3240

††These authors contributed equally: Yi-Chuan Tseng and Sih-Wei Chang

**Supplementary Material S1: Spatial distribution of energy density of electric dipole resonance of 120 nm Si sphere.**

**Figure S1.** Spatial distribution of energy density in a Si sphere having a diameter of 120 nm at wavelength of 430 nm.

**Supplementary Material S2: Optimization of thin-film cavity for a 90 nm Si sphere.**

Having decreased the diameter of the shrunken Si sphere to approximately 90 nm, we designed a new suitable thin-film cavity substrate to enhance the magnetic dipole resonance inside such a Si sphere. The simulated integrated energy density is shown in Figure S2b. An optimized thin-film cavity shows an integrated energy density with a maximum value of 6.52×10^-20^ μm^3^ when the SiO_2_ thickness (t_SiO2_) is 70 nm.

**Figure S2.** (a) Schematic representation of a Si sphere on a thin-film cavity substrate having a SiO_2_ film of various thicknesses. (b) Simulated integrated energy density of a Si sphere (diameter: 90 nm) placed upon thin-film cavity substrates having SiO_2_ layers with various thicknesses.

**Supplementary Material S3: Raman spectrum of Si spheres**

We used micro-Raman spectroscopy to characterize the crystallinity and quality of the Si spheres. Figure S3 displays the Raman spectra of the Si spheres before (120 nm) and after (90 nm) the thermal oxidation and wet etching processes. The Raman peaks of both Si spheres were located at almost the same position (520.6 cm^–1^) with almost the same full width at half maximum (FWHM = 9.34 cm^–1^), although their peak intensities were different; in addition, both Raman spectra featured symmetrical peaks before and after the size reducing process. We suspect that the lower intensity of signal from the Si sphere having a diameter of 90 nm was due to its lower volume. According to the Raman spectra, the Si spheres remained in the form of single-crystalline Si of the same quality after processing. Moreover, our analysis implied that the light emission in this study arose from the good quality of the crystalline Si spheres.

**Figure S3.** Raman spectra of Si spheres having diameters of 120 and 90 nm.

**Supplementary Material S4: The effect of oblique incidence**

To understand the mechanism of the thin-film cavity coupled with Si spheres, we simulated the integrated energy density of a 120 nm Si sphere placed on a thin-film cavity (80 nm SiO_2_/Ag) under oblique illumination. These results are shown in Figure S4.


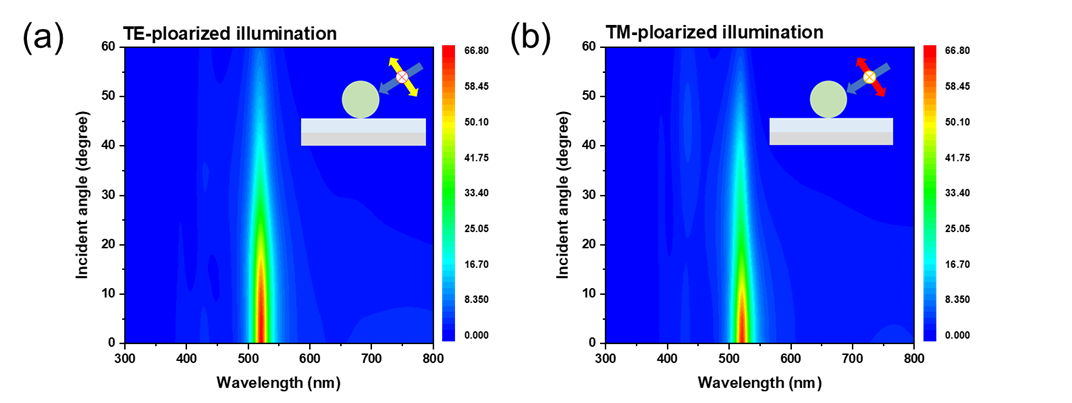


**Figure S4.** Si sphere (diameter=120 nm) placed on thin-film cavity (80 nm SiO_2_/Ag) under oblique illumination with (a) TE-polarization and (b) TM-polarization.

Figure S4 (a) shows the integrated energy density under a TE-polarized illumination. The integrated energy density doesn’t differ much when the incident angle is lower than 15^o^. However, when the incident angle increases to 15^o^, the integrated energy density further decreases and becomes much weaker than the normal incidence condition. The negative correlation between incident angle and integrated energy density can be explained by the co-enhancement effect from the magnetic dipole resonance of the Si sphere and the interference effect of the thin-film cavity. When the incident angle increases, the constructive-interference condition of the thin-film cavity is different, and hence a lower integrated energy density is obtained. In addition, with a TM-polarized illumination, the integrated energy density also shows a similar behavior (Figure S4(b)). The integrated energy density negatively correlates with the incident angle and is not weakened significantly until the incident angle is > 10^o^. The field confinement induced by the plasmonic effect under TM-polarized illumination has not been observed. These results show that the thin-film cavity is dominated by the interference effect rather than the plasmonic effect of Ag.

**Supplementary Material S5: Magnetic dipole resonance of elliptic Si**

The as-received Si spheres are not possible to be perfect spheres. The PAHL spectrum might be broadened relative to the simulation results. The SEM image of the as-received Si sphere is shown in Figure S5.


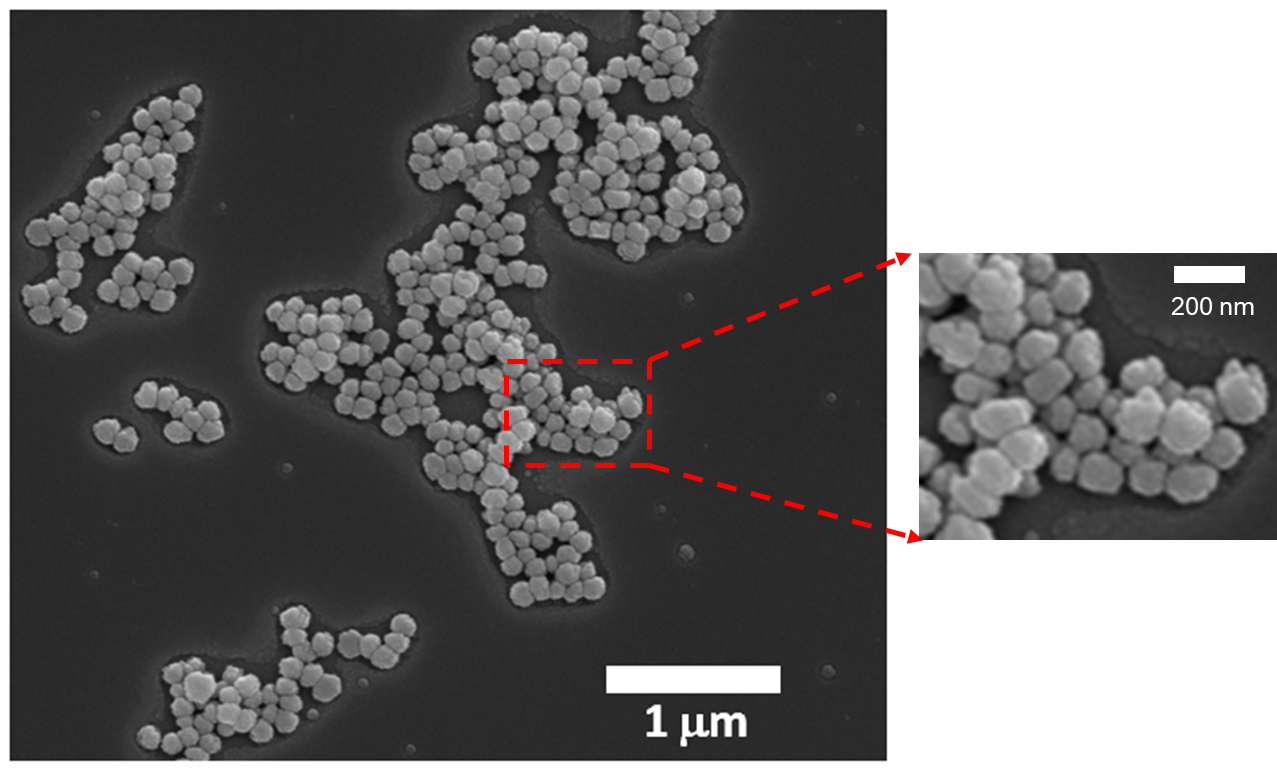


**Figure S5.** Top-view SEM image of the as-received Si sphere placed on Si substrate.

Top-view SEM image shows that Si spheres are not perfectly spherical. The Si spheres show an ellipsoid-like geometry, which can be utilized to explain the broadened spectra in the experiment. Hence, we measured the length of the long-axis and short-axis and calculated the (long-axis/ short-axis) ratio using ImageJ.

**Figure S6.** Histogram of (a) long-axis, (b) short-axis, and (c) long- to short-axis ratio.

As shown in Figure S6(a-b), the long- and short-axis length distribution shows a similar peak position around 115~125 nm. We found ca. 90% of the spheres have a long- to short-axis ratio between 1 to 1.3, and ca.10% have a long- to short-axis ratio larger than 1.3. The shapes may affect the field confinement of Si spheres. To further explain the effect of shape on the magnetic dipole resonance, we simulated the integrated energy of elliptic Si spheres on the thin-film cavity. The results are shown below in Figure S7.


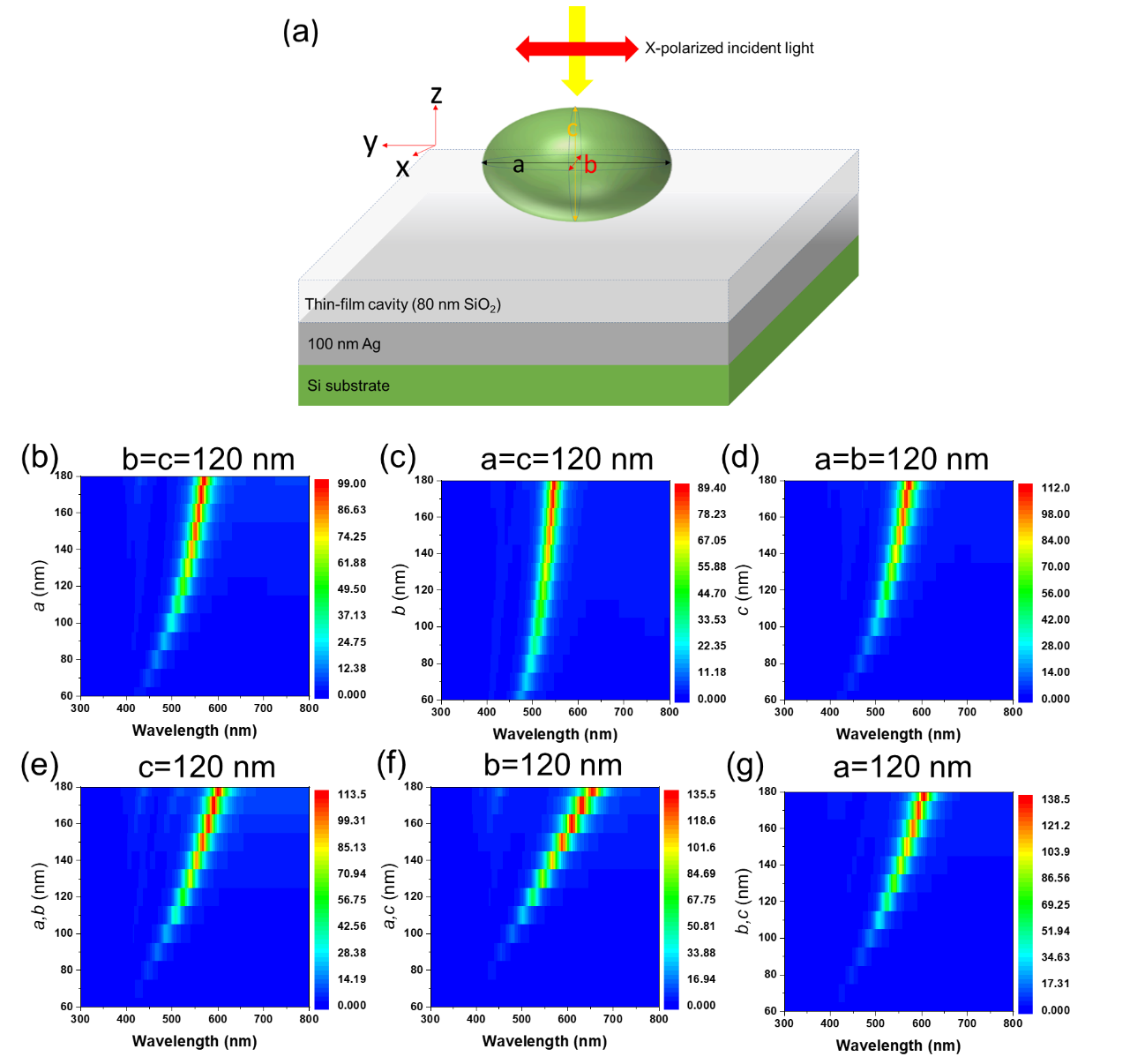


**Figure S7.** (a) Schematic representation of simulation of elliptic Si spheres. (b-d) Integrated energy density when lengths of *a*, *b*, and *c* are varied, respectively. (e-g) Integrated energy density when lengths of *a*, *b*, and *c* are fixed, respectively. The unit of the color bar is 10^-20^ um^3^.

For an elliptic Si sphere, the diameter along the x-, y-, and z-axis can be defined as *a*, *b*, and *c* (Figure S7(a)). We simulated the integrated energy density of elliptic Si spheres while two of the diameters were fixed and one of them was varied. Figure S7(b) shows the fixed *b* = *c* =120 nm and varied *a* from 60 to 180 nm. When ***a*** increases, the peak of energy density shows redshifts, which can be explained by the displacement loop induced by the magnetic dipole resonance. With a linear x-polarized light illumination, the direction of magnetic dipole moment is along the y-direction. The displacement current loop would lie on the x-z plane. When ***a*** increases, the increased perimeter of the displacement current loop can result in the accumulated phase in the Si sphere and a redshift in the resonance peak. We further simulated Si ellipsoid when the length of ***b*** or ***c*** was varied. Figure S7(c) shows that ***b*** (perpendicular to the polarization direction) varies from 60 to 180 nm. The peak of integrated energy density was also redshifted slightly. The increased length can explain the smaller redshift out of the x-z plane. When ***b*** increases, the perimeter of the circular displacement loop doesn't change and results in a small phase accumulation. However, when ***c*** varied, the significant redshift happens again. This redshift further confirms that the peak position of magnetic dipole resonance is affected by the perimeter of the circular displacement loop.

Additionally, we further simulate the integrated energy density of elliptic Si with one fixed diameter and the others varied. The results are shown in Figure S7(e-g). No matter which length of a diameter is fixed, the redshifts can be observed. Especially for the case of fixed ***b***, the integrated energy density shows the most significant redshift. This result confirms that the phase accumulation along the x-, and z-axis is significant. In summary, the magnetic dipole resonance of the Si sphere still exists when the geometry is not a strict sphere. Even with a non-spherical Si, the high refractive index of Si can induce a strong displacement current loop.

**Supplementary Material S6: The size distribution of Si spheres**

To explain the peak difference in Figure 5(c, d), we measured the size distribution of the Si spheres according to the SEM image and calculated the size-weighted integrated energy density. The SEM image and size distribution are shown in Figure S8. The size-weighted integrated energy density can be calculated through the following formula:

$$\left\langle\bar{\rho} \right\rangle=\frac{\int\left\langle\rho\left( D \right) \right\rangle N(D)dD}{\int N(D)dD}$$

where $\left\langle\bar{\rho} \right\rangle$, $\left\langle\rho\left( D \right) \right\rangle$, *N*(*D*), and *D* are the size-weighted integrated energy density, integrated energy density, number distribution of size, and diameter, respectively. *N*(*D*) is according to the size distribution measured by SEM. A Gaussian distribution is utilized to fit the measured size distribution. As shown in Figure S9, the size-weighted integrated energy density shows good predictions of the PHAL signals. The size distribution of Si spheres can result in a broader peak. It can be noted that a higher simulated integrated energy density than the measured PHAL signals in the longer wavelength region. The reason for this overestimation might be phonon collision when excitons interact with phonons. Due to the indirect bandgap of Si, the emission of lower photon energy needs a multi-phonon interaction, which results in lower emission intensity.

**Figure S8.** SEM images of Si spheres with sizes ranging from (a) 115~125 nm, (b) 105~115 nm, (c) 95~105 nm, and (c) 85~95 nm, respectively.


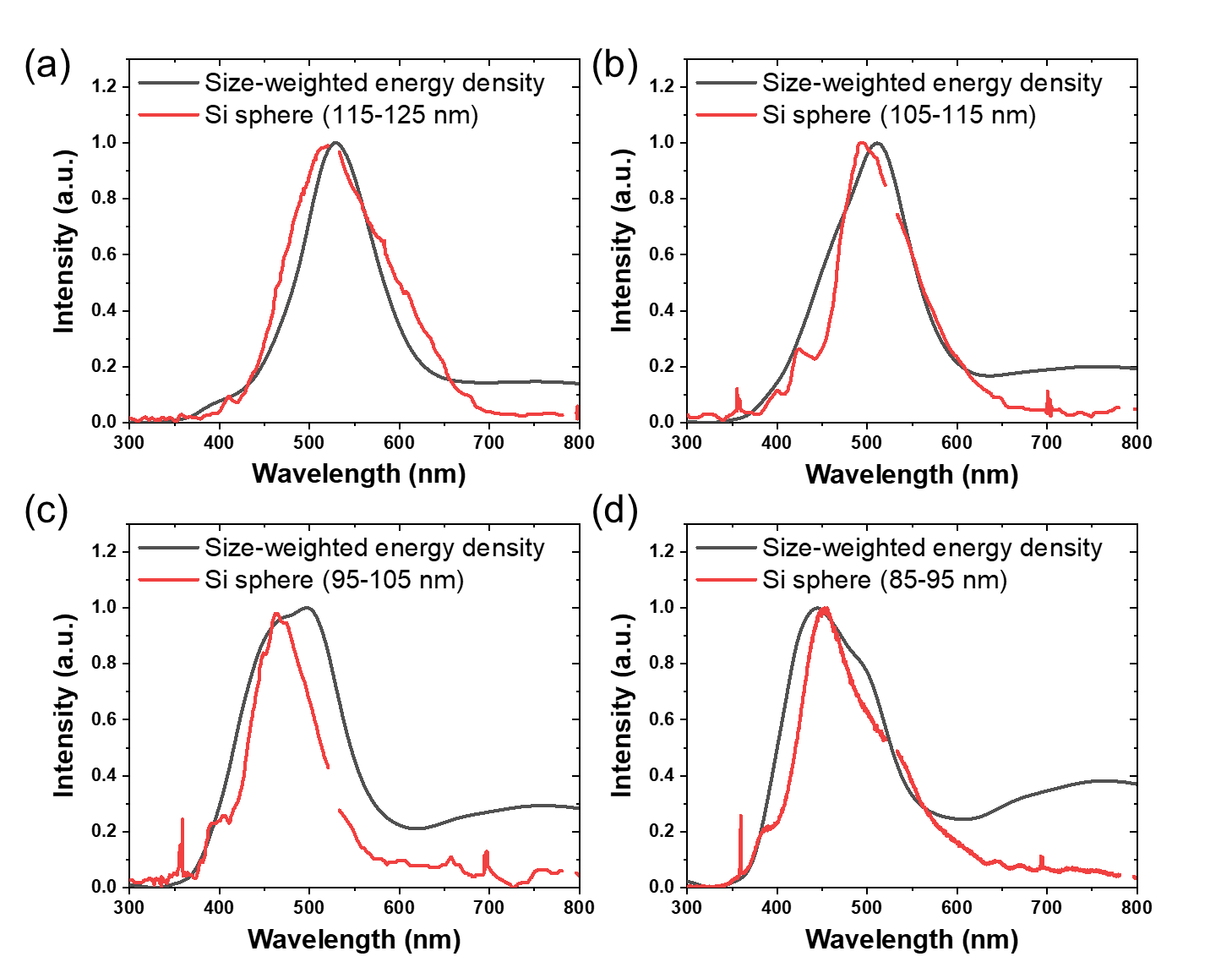


**Figure S9.** Measured PAHL intensity and calculated size-weighted integrated energy density of Si spheres having diameters of (a)115-125 nm, (b) 105-115 nm, (c) 95-105 nm, and (d) 85-95 nm.

**Table S1** Characteristics of the light emitted from Si QDs and Si spheres having different diameters, excitation wavelengths, peak wavelengths, and peak sensitivities.

| **Emitter** | **Diameter (nm)** | **Peak (nm)** | **Sensitivity (△λ_peak_/△D)** | **Ref. (Year)** |
| --- | --- | --- | --- | --- |
| Si QDs | 2.5 | 736.7 | 61.6 | 3 (2010) |
|  | 3.0 | 767.5 | 81.9 |  |
|  | 3.5 | 808.4 | 89.8 |  |
|  | 4.0 | 853.3 | 48.7 |  |
|  | 4.5 | 877.7 | 101.3 |  |
| Si QDs | 2.7 | 718 | 925.0 | 4 (2012) |
|  | 2.9 | 903 | 25.7 |  |
|  | 5.0 | 957 | 29.0 |  |
|  | 6.0 | 986 | 24.5 |  |
| Si QDs | 1.9 | 609.4 | 100.5 | 5 (2017) |
|  | 2.1 | 629.5 | 66 |  |
|  | 2.3 | 642.7 | 41 |  |
|  | 2.5 | 650.9 | 260 |  |
|  | 2.6 | 676.9 | 140.5 |  |
|  | 2.8 | 705.0 | 306 |  |
|  | 2.9 | 735.6 | 71 |  |
| Si QDs | 3.1 | 711.1 | 115 | 6 (2020) |
|  | 3.3 | 734.1 | 76 |  |
|  | 3.6 | 756.9 | 58 |  |
|  | 3.9 | 774.3 | 104 |  |
|  | 4.1 | 795.1 | 87 |  |
|  | 4.0 | 786.4 | 67.75 |  |
| Si QDs | 1.20 | 530.2 | 323.3 | 7 (2020) |
|  | 1.23 | 539.9 | 333.3 |  |
|  | 1.26 | 549.9 | 460 |  |
|  | 1.3 | 568.3 | 26 |  |
|  | 1.5 | 573.5 | 76 |  |
|  | 1.8 | 596.3 | 487 |  |
|  | 1.9 | 645.0 | 329.5 |  |
|  | 2.1 | 710.9 | 98.6 |  |
| Si sphere | 90 | 452 | 1.0 | This work |
|  | 100 | 462 | 3.2 |  |
|  | 110 | 494 | 2.6 |  |
|  | 120 | 520 | 4.3 |  |

**Reference**

(1) Albert, M. B., Standards for photoluminescence quantum yield measurements in solution (IUPAC Technical Report). *Pure Appl. Chem.* **2011,** *83* (12), 2213-2228.

(2) Lu, D.; Kan, J. J.; Fullerton, E. E.; Liu, Z., Enhancing spontaneous emission rates of molecules using nanopatterned multilayer hyperbolic metamaterials. *Nat. Nanotechnol.* **2014,** *9* (1), 48-53.

(3) Pringle, T. A.; Hunter, K. I.; Brumberg, A.; Anderson, K. J.; Fagan, J. A.; Thomas, S. A.; Petersen, R. J.; Sefannaser, M.; Han, Y.; Brown, S. L., Bright Silicon Nanocrystals from a Liquid Precursor: Quasi-Direct Recombination with High Quantum Yield. *ACS Nano* **2020,** *14* (4), 3858-3867.

(4) Shirahata, N.; Nakamura, J.; Inoue, J.-i.; Ghosh, B.; Nemoto, K.; Nemoto, Y.; Takeguchi, M.; Masuda, Y.; Tanaka, M.; Ozin, G. A., Emerging Atomic Energy Levels in Zero-Dimensional Silicon Quantum Dots. *Nano Lett.* **2020,** *20* (3), 1491-1498.

(5) Yu, Y.; Fan, G.; Fermi, A.; Mazzaro, R.; Morandi, V.; Ceroni, P.; Smilgies, D.-M.; Korgel, B. A., Size-dependent photoluminescence efficiency of silicon nanocrystal quantum dots. *J. Phys. Chem. C* **2017,** *121* (41), 23240-23248.

(6) Hessel, C. M.; Reid, D.; Panthani, M. G.; Rasch, M. R.; Goodfellow, B. W.; Wei, J.; Fujii, H.; Akhavan, V.; Korgel, B. A., Synthesis of Ligand-Stabilized Silicon Nanocrystals with Size-Dependent Photoluminescence Spanning Visible to Near-Infrared Wavelengths. *Chem. Mater.* **2012,** *24* (2), 393-401.

(7) de Boer, W. D. A. M.; Timmerman, D.; Dohnalová, K.; Yassievich, I. N.; Zhang, H.; Buma, W. J.; Gregorkiewicz, T., Red spectral shift and enhanced quantum efficiency in phonon-free photoluminescence from silicon nanocrystals. *Nat. Nanotechnol.* **2010,** *5* (12), 878-884.
